# Spatio-Temporal Data Comparisons for Global Highly Pathogenic Avian Influenza (HPAI) H5N1 Outbreaks

**DOI:** 10.1371/journal.pone.0015314

**Published:** 2010-12-20

**Authors:** Zhijie Zhang, Dongmei Chen, Yue Chen, Wenbao Liu, Lei Wang, Fei Zhao, Baodong Yao

**Affiliations:** 1 Department of Geography, Queen's University, Kingston, Canada; 2 Department of Epidemiology, Fudan University, Shanghai, People's Republic of China; 3 Department of Epidemiology and Community Medicine, University of Ottawa, Ottawa, Canada; Dana-Farber Cancer Institute, United States of America

## Abstract

Highly pathogenic avian influenza subtype H5N1 is a zoonotic disease and control of the disease is one of the highest priority in global health. Disease surveillance systems are valuable data sources for various researches and management projects, but the data quality has not been paid much attention in previous studies. Based on data from two commonly used databases (Office International des Epizooties (OIE) and Food and Agriculture Organization of the United Nations (FAO)) of global HPAI H5N1 outbreaks during the period of 2003–2009, we examined and compared their patterns of temporal, spatial and spatio-temporal distributions for the first time. OIE and FAO data showed similar trends in temporal and spatial distributions if they were considered separately. However, more advanced approaches detected a significant difference in joint spatio-temporal distribution. Because of incompleteness for both OIE and FAO data, an integrated dataset would provide a more complete picture of global HPAI H5N1 outbreaks. We also displayed a mismatching profile of global HPAI H5N1 outbreaks and found that the degree of mismatching was related to the epidemic severity. The ideas and approaches used here to assess spatio-temporal data on the same disease from different sources are useful for other similar studies.

## Introduction

Avian influenza virus (AIV) is one type of *Orthomyxovirus* with a genome of eight single-stranded, negative sense RNA segments encoding 11 proteins (PB2, PB1, PB1-F2, PA, HA, NP, NA, M1, M2, NS1, and NS2), which can be classified into different subtypes according to envelope proteins of HA and NA [1]–[2]. AIV that infects domestic poultry can be roughly divided into two groups based on disease severity, highly pathogenic avian influenza (HPAI) and low pathogenic avian influenza (LPAI) [3]. The flock mortality is as much as 100% for HPAI (mainly H5 and H7 subtypes). There has been a great threat of potential influenza pandemic from HPAI H5N1 since late 2003 [4], which has posed major challenges to both human health and poultry industry [5] and is considered as one of the highest priority diseases in the last decade.

As a transboundary animal disease (TAD), HPAI H5N1 has a potential capability of serious and rapid spreading triggered by bird migration [6]–[8] and international poultry trades [9], but irrespective of national boundaries. This gives high prominence to global disease surveillance systems and international collaborations for fighting TADs. Several international organizations have devoted to collect and track global HPAI H5N1 outbreaks, including the Food and Agriculture Organization of the United Nations (FAO), Office International des Epizooties (OIE), World Health Organization (WHO), and the European Union (EU) [10]–[11]. These global surveillance systems provide valuable data sources for HPAI H5N1 researches [12]–[14]. Two most frequently used global HPAI H5N1 databases are FAO EMPRES-i and OIE WAHID. For example, Gilbert et al. used FAO data between 2005 and 2006 to discuss the spread of HPAI H5N1 in the Western Palearctic [6]; Si et al. used the OIE data during 2003 to 2006 to study the relationships between spatio-temporal dynamics of global H5N1 outbreaks and bird migration patterns [15]; OIE data from 2007 and 2008 were used by Ahmed et al. to analyze spatio–temporal clustering of HPAI H5N1outbreaks in Bangladesh [16]; Cecchi et al. used data from both FAO and OIE for the period of 2006–2007 for different illustrations in their study [17] and found some differences between two databases, but provided no extensive discussion. Different data sources may result in different and sometimes conflicting results in the area. Data quality becomes a concern, but has not been previously assessed.

This study used OIE and FAO databases to examine their temporal, spatial and spatio-temporal patterns of global HPAI H5N1 outbreaks. Through detailed comparisons on the two databases, we provided some useful ideas and approaches for comparing spatio-temporal data on the same disease from different sources.

## Materials and Methods

### 1. Data collection

#### 1.1 FAO EMPRES-i data

Working with affected and at-risk countries for capacity building, information sharing and networking construction since December 2003, FAO has nowadays provided control and preparedness support of HPAI H5N1 to 95 countries. The Emergency Prevention System for Transboundary Animal and Plant Pests and Diseases (EMPRES) was established in 1994 to support the early warning and reaction component and a web-based EMPRES Global Animal Disease Information System (EMPRES-i) was designed to gather and share the information on major TADs such as HPAI H5N1(http://www.fao.org/). EMPRES-i collects disease information from various sources including official organization (e.g. WHO, OIE and European Commission), unofficial organizations (e.g. country or regional project reports and field mission reports), and others such as media-based reports and disseminated reports (e.g. the Program for Monitoring Emerging Diseases (ProMed) and Global Public Health Intelligence Network (GPHIN)). All information is entered into the EMPRES-i database after careful checking [11]. Each outbreak has been attached many attributes including spatio-temporal information (observation date and geo-referenced location with corresponding names of administrative units). Data on confirmed domestic H5N1 outbreaks from January 1, 2004 to December 31, 2009 were obtained from EMPRES-i. If multiple outbreaks appeared in the same day and the same smallest administrative unit, then they were merged into one outbreak. Data of HPAI H5N1 outbreaks before 2004 were not available in EMPRES-i. An earlier version of FAO dataset for the period from December 1, 2003 to December 15, 2005 was provided by Dr. Declan Butler, which was used to generate the first online Google Earth map of global H5N1outbreaks and was accompanied with two articles in Nature [18]–[19]. By combining the above two databases, we created a more complete FAO dataset for the period from December 1, 2003 to December 31, 2009.

#### 1.2 OIE WAHID data

The Office International des Epizooties (OIE) was created in Paris in 1924 by 28 countries and became the World Organization for Animal Health in May 2003 while its historical acronym OIE was kept. In 2010, it has a total of 176 member countries and territories, and maintains permanent relationships with 36 other international and regional organizations (http://www.oie.int/eng/en_index.htm).

Since December 2003, OIE has been tracking global HPAI H5N1 outbreaks. All information on reported HPAI H5N1 outbreaks is obtained from the World Animal Health Information Database (WAHID) in PDF files. For the period from January 2006 to December 2009, HPAI H5N1 reporting tables in PDF files provided outbreaks information which was entered twice into an EXCEL spreadsheet and then consistency checking was conducted. The reporting tables, however, were not available for the period from December 2003 to December 2005.Those outbreak information had to be extracted from related articles manually. Details about the process of information extraction and correction are available upon request.

### 2. Data matching and integration

An H5N1 outbreak was defined as a confirmed presence of the disease, clinically expressed or not, in at least one individual in a defined administrative unit in one day [20]. During the data processing, we first merged multiple outbreak records for the same day and the same subdistrict and considered them as one outbreak for both OIE and FAO data. The coordinates for the subdistrict's centorid were used to represent the merged outbreak. The matching of two databases was performed based on temporal and spatial attributes of an outbreak, which were indicated by observation/outbreak date and five administrative units of country, province, district, subdistrict and precise location. Subdistrict was the smallest matching unit in this study. The matching results were indicated by two new variables in the FAO dataset. One variable recorded the key variable of matched outbreak from OIE data and another variable of three categories described the matching degree: “C” - complete match for both space and time attributes; “A” - complete match of space attribute and time difference ≤7 days; “N” - spatial attributes not matched or temporal difference >7 days or both. This process was completed manually and the matching results were thrice checked. Finally, OIE and FAO data were merged into an integrated OIE-FAO database for global HPAI H5N1 outbreaks, which includes four subsets of completely matched, almost matched, and unmatched HPAI H5N1 outbreaks from OIE and FAO, respectively.

### 3. Statistical analysis

Descriptive statistics were first used to summarize the matching results for the OIE and FAO datasets. Then day-based time-series plots were generated for visual comparison on temporal patterns of global HPAI H5N1 outbreaks. To measure temporal (dis)similarity quantitatively, HPAI H5N1 outbreaks were aggregated into weekly counts and analyzed using Wilcoxon signed rank test for two dependent samples and Spearman rank correlation analysis, respectively.

For mapping spatial distribution of global HPAI H5N1 outbreaks, the centroids of subdistrict-based units were used. To quantitatively assess their differences across space, the outbreaks were aggregated into counts based on province and then Wilcoxon signed rank test for two dependent samples and Spearman rank correlation analysis were applied to determine their spatial (dis)similarity. To explore it further, smoothing maps for matched and unmatched outbreaks were generated for comparing their differences. The number of unmatched outbreaks from FAO data divided by the number of all OIE outbreaks was calculated to represent the mismatching conditions in each country and pie charts with each year's mismatching data as pie components were generated and overlaid over the country to show global mismatching situations.

Finally, two spatio-temporal analysis approaches were used to evaluate the agreement between spatio-temporal patterns generated from OIE and FAO data: spatio-temporal *K* function (st*K*) and spatial-temporal multi-response permutation procedure (stMRPP). st*K* was a generalization of Ripley's two-dimensional *K* function [21] and defined as
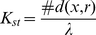
(1)Where, *λ* is the theoretical intensity of the spatio-temporal process that is the expected number of points per unit volume and *#d*(*x,r*) is the number of observed points, excluding *x* itself, which fall within a distance *r* of *x* in three-dimensional space. The three-dimensional version of Ripley's isotropic edge correction was applied to correct boundary effects [21]–[22].

*D_st_* statistics, following the ideas of two-dimensional cases [16], [23], was used based on st*K*, to examine the dissimilarity in the spatio-temporal patterns:
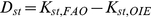
(2)The theoretical values of *D_st_* is 0 if OIE and FAO data provide the same spatio-temporal distributions. Monte Carlo permutation procedure was used to generate simulated datasets and envelope of 95% confidence interval (eCI) for assessing the significance of *D_st_*. If the observed *D_st_* was located inside eCI, no significant difference was found. Otherwise, significant difference was detected.

stMRPP was calculated by the following formula [24]–[25],
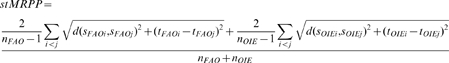
(3)Where, the spatial position and outbreak date from OIE and FAO data were represented by (*s*_FAO*i*_, *t*_FAO*i*_), *i* = 1, …, *n*_FAO_ and (*s*_OIE*i*_, *t*_OIE*i*_), *i* = 1, …, *n*_OIE_, respectively; *d*(*s*_FAOi_, *s*_FAOj_) and *d*(*s*_OIEi_, *s*_OIEj_) were used to represent the Euclidean distances between locations of *s*_FAOi_ and *s*_FAOj_, and *s*_OIEi_and *s*_OIEj_, respectively. Significance test was based on Monte Carlo permutation procedures. First, *N* simulated datasets for OIE and FAO were generated and then simulated stMRPP statistics were computed for the simulated data using formula (3). Finally, *p*-value was obtained by (*M*+1)/(*N*+1) (*M* is the rank of observed stMRPP statistic among simulated stMRPP statistics; *N* is the number of simulations).

## Results

### 1. Data summaries

Table 1 shows that FAO captures more HPAI H5N1 outbreaks in 2004 and 2009 than OIE, but fewer outbreaks in other years. The degree of missing was substantial for 2005/2006 and then decreased gradually, which can be seen from the number of unmatched OIE outbreaks and the values of missing proportion (MP). Besides, there are a large amount of outbreaks in 2004 for FAO.

**Table 1:** Matching results of global HPAI H5N1outbreaks for the FAO and OIE datasets: 2003 to 2009.

Year	Match degree				Total			MP*
	A	C	N		OIE	FAO	OIE+FAO	
			OIE	FAO				
2003†	0	3	12	0	15	3	15	0.8
2004	410	124	313	3436	847	3970	4283	0.36954
2005	60	171	634	71	865	302	936	0.732948
2006	171	315	858	305	1344	791	1649	0.638393
2007	138	292	184	110	614	540	724	0.299674
2008	47	381	94	55	522	483	577	0.180077
2009	11	59	29	144	99	214	243	0.292929
Total	837	1345	2124	4121	4306	6303	8427	0.493265

*MP is the proportion of OIE data missed by FAO, or the number of unmatched outbreaks in OIE divided by the total number of outbreaks in OIE;

† only includes HPAI H5N1 outbreaks in December, 2003.

### 2. Time-series plots of global HPAI H5N1 outbreaks

**Figure 1:**
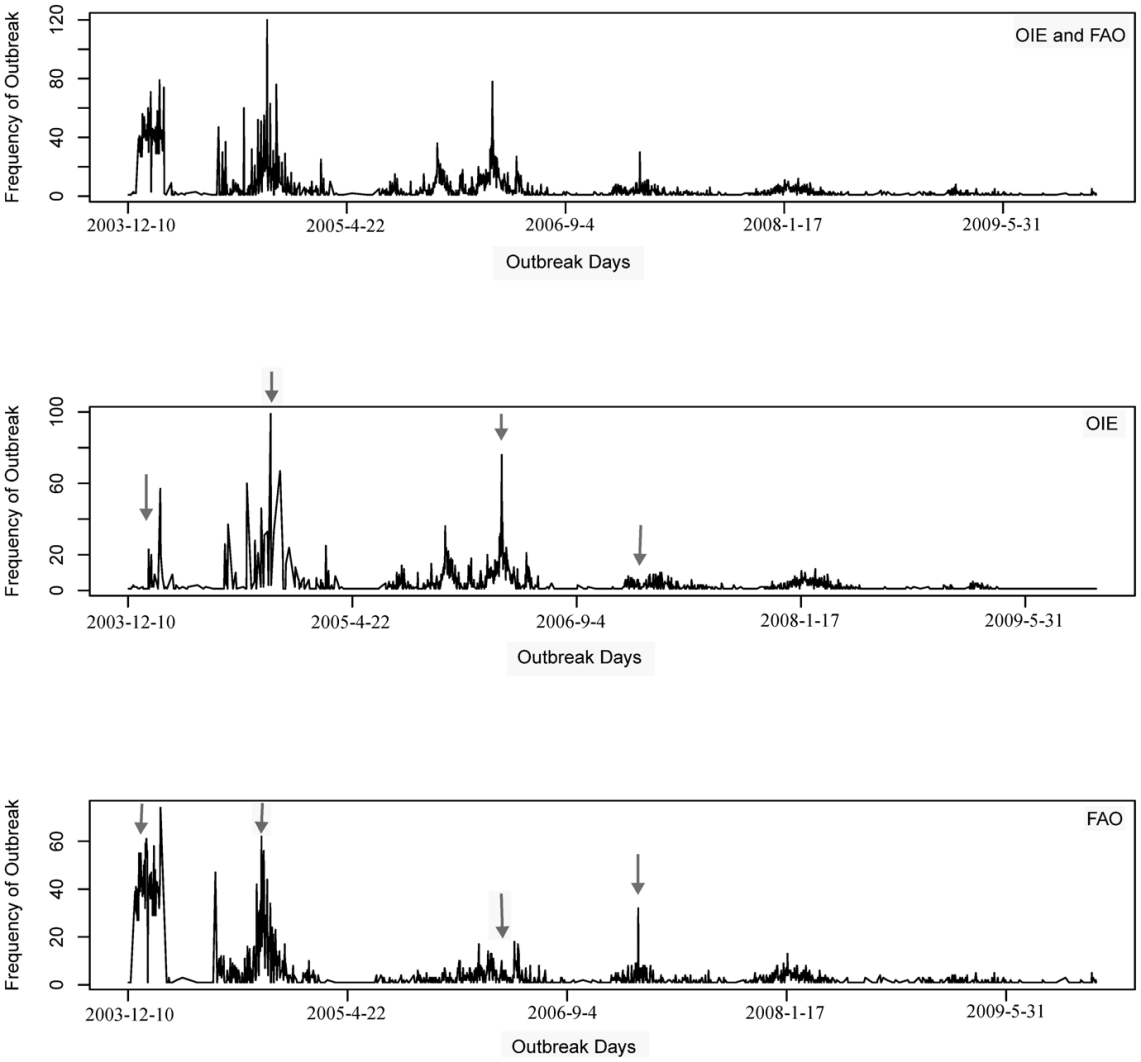
Time series plots of global HPAI H5N1 outbreaks. Generally speaking, OIE and FAO reflect similar temporal patterns. But, the discrepancies are also obvious in the relatively detailed temporal pattern such as those places highlighted by arrows.

OIE and FAO data showed similar temporal trends for HPAI H5N1 outbreaks in general, but there were some discrepancies. For example, temporal patterns in around 2003-12-29, 2004-10-4, 2006-5-27 and 2007-2-1 show different shapes (Figure 1). However, Wilcoxon signed rank test did not detect a significant difference between OIE and FAO data (*V = 16366*, *p = 0.52*). Spearman rank correlation analysis showed a medium degree of correlation (*r* = 0.73, *p*<0.0001).

### 3. Spatial distribution of global HPAI H5N1 outbreaks

**Figure 2:**
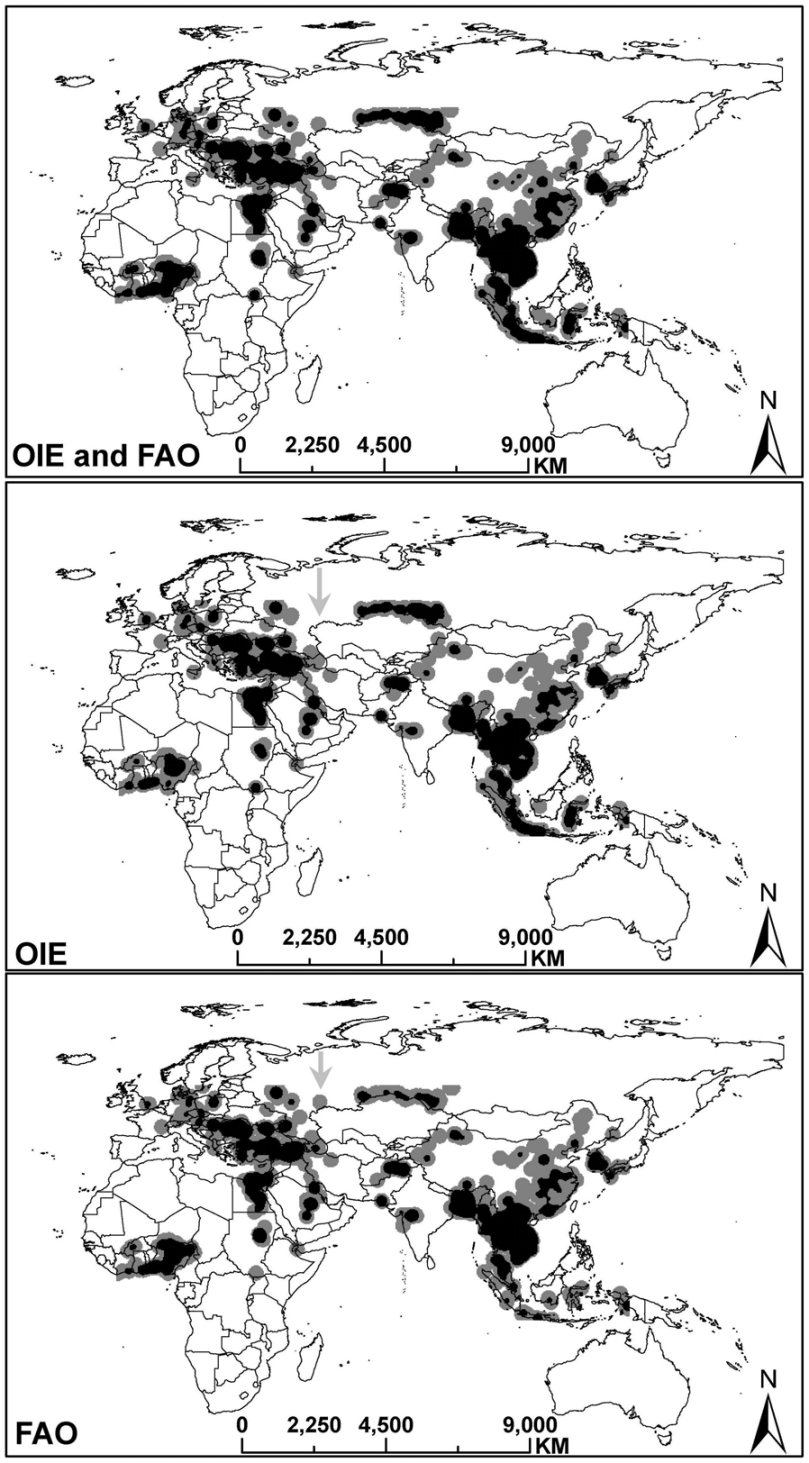
Spatial distribution of global HPAI H5N1 outbreaks. A high degree of agreement on spatial pattern is obvious between OIE and FAO from a qualitative perspective. However, some minor differences can also be seen such as the middle part of the maps highlighted in arrows.

Figure 2 depicts the spatial distribution of global HPAI H5N1 outbreaks from OIE and FAO. From a qualitative perspective, there is a medium degree of agreement in spatial patterns between the two datasets, which was confirmed by Spearman rank correlation analysis (*r* = 0.79, *p*<0.0001). But minor differences still existed such as the middle part of the map although no significant differences were found by Wilcoxon signed rank test (*V* = 11474.5, *p* = 0.14).

**Figure 3:**
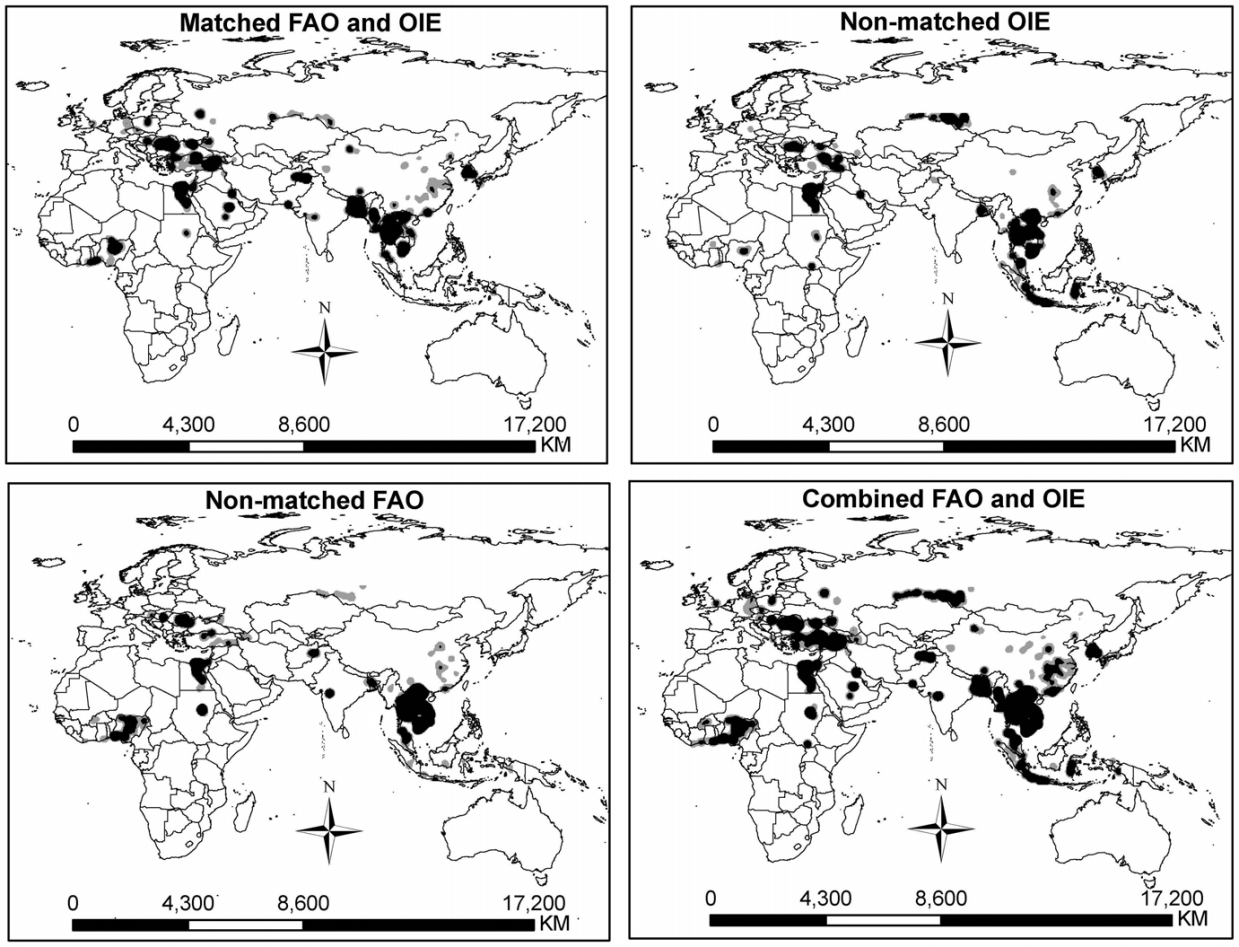
Spatial distributions of matched and unmatched H5N1 outbreaks for OIE and FAO data. The spatial distributions of unmatched outbreaks are similar and they are also similar with the distribution of matched outbreaks. This prompts that the general spatial patterns captured by individual OIE and FAO are similar, but the quantitative information recorded by them are different.

Figure 3 shows that unmatched outbreaks from OIE and FAO appeared to have a similar spatial distribution compared with matched outbreaks and the more reported cases in a region, the more unmatched outbreaks.

**Figure 4:**
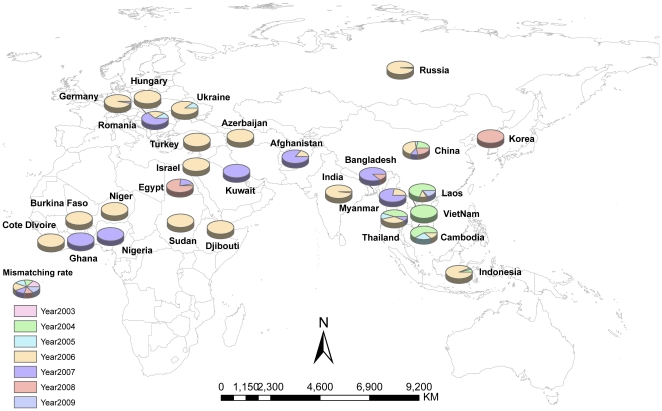
Country-based mismatching profile of global HPAI H5N1 outbreaks. In 2003/2004, the mismatching cases were only located in the Southeast Asia; in 2005 the mismatching outbreaks began to appear in Europe such as Ukraine and Romania; in 2006, the mismatching situations further spread to the Africa and reached a peak from the spatial perspective; in 2007 and thereafter, the mismatching situation began to mitigate gradually. This mismatching profile seems to be consistent with the global epidemic situation of HPAI H5N1 outbreaks.

Figure 4 shows the information on the mismatching situation of global HPAI H5N1 outbreaks at the country level. In 2003/2004, the mismatched cases were confined in the Southeast Asia; in 2005 the mismatched outbreaks began to appear in Europe (i.e., Ukraine and Romania); in 2006, the mismatched situations spread further to Africa and reached a peak from the spatial perspective; in 2007 and thereafter, the mismatched situation started to mitigate gradually.

### 4. Spatio-temporal consistency test

**Figure 5:**
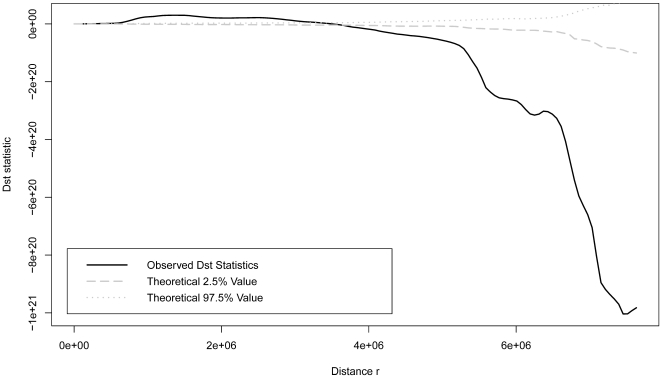
Results of Spatial-Temporal *K*-function analysis. OIE and FAO data have significantly different spatio-temporal distributions because the observed Dst statistic is outside the envelope of 95% confidence interval. When the observed distance is ≤4e+06m, the FAO data is more clustered than OIE data, but more regular than OIE data if the study scale is >4e+06m.

The results of st*K* show that OIE and FAO data were significantly different for their spatio-temporal distributions. When the observed scale is ≤4e+06 m, FAO data seemed to be more clustered than OIE data, but it reversed when the observed scales >4e+06 m (Figure 5). And stMRPP also identified distinguished differences on their spatio-temporal distribution (stMRPP = 2.05, *p*<0.01).

## Discussion

HPAI H5N1 is a zoonotic disease with serious impacts on poultry industry, wildlife, economics, and public health [26]. To control the disease, global disease surveillance systems play an important role [10]–[11]. Many published studies of HPAI H5N1 have used different data sources and have assumed that these data are valid and reliable. Data assessment is always an important first step if the data from disease surveillance systems are used for inference-based analysis [12]–[13], [27]–[28], but no studies of data evaluation have previously been conducted in the area of HPAI H5N1 research. There is a need for an improvement of the data completeness (personal communications with Drs. Julio Pinto and Daniel Beltran-Alcrudo from the FAO global early warning system). In this study, we conducted detailed spatio-temporal data comparisons on two commonly used datasets of OIE and FAO.

The comparison results show that there were obvious differences between OIE and FAO data and prompt that an integrated dataset of OIE and FAO may provide improved information of global H5N1 outbreaks. The most number of H5N1 outbreaks seems to appear in 2004, when FAO includes about 4000 outbreaks which were mainly from Thailand and Viet Nam (over 1500 and 2000 outbreaks, respectively). However, they both reported less than 10 outbreaks in 2006, when more countries reported H5N1 outbreaks. So a more reasonable statement for global HPAI H5N1 may be that the outbreaks started from late 2003, peaked in number in 2004 and reached the serious epidemic in 2006 from the spatial perspective. There were outbreaks recorded in OIE data were missed in FAO data and the missing degree tended to be in line with epidemic situation of global HPAI H5N1 outbreaks, which was most serious for 2005/2006 and then decreased gradually.

When we consider the temporal and spatial patterns of global HPAI H5N1 outbreaks separately, data from OIE and FAO tended to have a similar pattern and medium concordance although some minor discrepancies existed. We also used two more advanced approaches to compare spatio-temporal patterns of global H5N1 outbreaks between OIE and FAO. stMRPP is a nonparametric approach that does not depend on arbitrarily assigned origins and not require any estimation or modeling of spatial correlation compared to traditional multiple response permutation procedures (MRPP). It has a higher statistical power through simulation studies [24]; st*K* is a parametric method, which can provide a comprehensive profile through analyzing the data across different scales. Both approaches led to the same conclusion that OIE and FAO data provided significantly different spatio-temporal distributions of global H5N1 outbreaks.

OIE and FAO are independent international organizations, and their collaborator/partner networks and information sources are not the same. Hence it is very possible that they may capture some complementary data on HPAI H5N1 outbreaks. Combining these two datasets would provide an improved database with more complete spatio-temporal outbreak information.

Clearly, underreporting of global HPAI H5N1 outbreaks is inevitable, and H5N1, as is the case with other animal disease, is commonly underreported across all types of production systems, particularly in the commercial poultry sectors in many countries [25], [27], [29]. Several reasons may result in this underreporting: 1) Active surveillance of H5N1 in countries where the disease is endemic is always low or absent, which makes the national surveillance systems do not have enough capacity to capture the whole profile of HPAI H5N1 outbreaks [25]; 2) Unwillingness to report H5N1 outbreaks due to reasons such as political pretexts [30]; 3) HPAI H5N1 is not a disease with high priority in some regions, so awareness of the need and importance to report outbreaks is lacking [31]; and 4) Stakeholders do not like to report H5N1 outbreaks because of inadequate compensation for culled animals if reported [32]. It is difficult to measure the accurate underreporting situation becaue of lacking gold standard, but the mismatching case may reflect the underreporting situation to an extent. Underreporting may lead to the difficulty of obtaining data, while different organizations have different ability to handle this issue, which can result in their data difference-mismatching. We demonstrated the mismatching profile of global HPAI H5N1 outbreaks over time that seemed to be related to the severity of epidemic situation. Several countries (e.g., Hungary, Israel and Turkey) had relatively high mistaching cases in 2006 because no outbreaks were reported in the other years. Since it is not possible to have a 100% complete dataset of global HPAI H5N1 outbreaks, we need to develop some methods to alleviate the potential impacts of underreporting for accurate analysis.

In summary, we conducted spatio-temporal comparisons between two commonly used datasets of global HPAI H5N1 outbreaks (OIE and FAO) from temporal, spatial and spatio-temporal point of views for the first time. Two datasets showed similar spatial and temporal distributions of outbreaks when they were considered separately, but more advanced methods detected a significant difference in the joint spatio-temporal distribution. Because of incompleteness for both OIE and FAO datasets, an integration of them would provide a more complete picture of global HPAI H5N1 outbreaks. The ideas and approaches for spatio-temporal data comparisons can be used in other similar studies. Future work will involve using the integrated dataset to explore long-term effects of control strategies on global HPAI H5N1 outbreaks, identify long-term variations in disease patterns and dynamics, detect potential mechanisms of driving its spread, conduct multi-scale analysis on various risk factors, and evaluate potential impacts of climate change, among others.

## References

[1] Nelson MI, Holmes EC (2007). The evolution of epidemic influenza.. Nature.

[2] Kaiser J (2006). A one-size-fits-all flu vaccine?. Science.

[3] Alexander DJ (2000). A review of avian influenza in different bird species.. Vet Microbiol.

[4] Alexander DJ (2007). Summary of avian influenza activity in Europe, Asia, Africa, and Australasia, 2002–2006.. Avian Dis.

[5] Capua I, Alexander DJ (2009). Avian influenza infection in birds: a challenge and opportunity for the poultry veterinarian.. Poult Sci.

[6] Gilbert M, Xiao X, Domenech J, Lubroth J, Martin V (2006). Anatidae migration in the western Palearctic and spread of highly pathogenic avian influenza H5NI virus.. Emerg Infect Dis.

[7] Liu J, Xiao H, Lei F, Zhu Q, Qin K (2005). Highly pathogenic H5N1 influenza virus infection in migratory birds.. Science.

[8] Chen H, Smith GJ, Zhang SY, Qin K, Wang J (2005). Avian flu: H5N1 virus outbreak in migratory waterfowl.. Nature.

[9] Birdlife International (2006). Illegal imports probable cause of Nigeria flu.. http://www.birdlife.org/news/news/2006/02/avian_flu_nigeria.html.

[10] Pittman M, Laddomada A, Freigofas R, Piazza V, Brouw A (2007). Surveillance, prevention, and disease management of avian influenza in the european union.. J Wildl Dis.

[11] Martin V, Dobschuetz SV, Lemenach A, Rass N, Schoustra W (2007). Early warning, database, and information systems for avian influenza surveillance.. J Wildl Dis.

[12] Henning J, Pfeiffer DU, Vu le T (2009). Risk factors and characteristics of H5N1 Highly Pathogenic Avian Influenza (HPAI) post-vaccination outbreaks.. Vet Res.

[13] Farnsworth ML, Ward MP (2009). Identifying spatio-temporal patterns of transboundary disease spread: examples using avian influenza H5N1 outbreaks.. Vet Res.

[14] Pfeiffer DU, Minh PQ, Martin V, Epprecht M, Otte MJ (2007). An analysis of the spatial and temporal patterns of highly pathogenic avian influenza occurrence in Vietnam using national surveillance data.. Vet J.

[15] Si Y, Skidmore AK, Wang T, de Boer WF, Debba P (2009). Spatio-temporal dynamics of global H5N1 outbreaks match bird migration patterns.. Geospat Health.

[16] Ahmed SS, Ersbøll AK, Biswas PK, Christensen JP (2010). The space-time clustering of highly pathogenic avian influenza (HPAI) H5N1 outbreaks in Bangladesh.. Epidemiol Infect.

[17] Cecchi G, Ilemobade A, Le Brun Y, Hogerwerf L, Slingenbergh J (2008). Agro-ecological features of the introduction and spread of the highly pathogenic avian influenza (HPAI) H5N1 in northern Nigeria.. Geospat Health.

[18] Butler D (2006). Mashups mix data into global service.. Nature.

[19] Butler D (2006). Nature gets mashed up: Technology helps to map bird flu around the globe.. Nature.

[20] Toma B, Vaillancourt JP, Dufour B, Eloit M, Moutou F (1999). Dictionary of Veterinary Epidemiology.

[21] Ripley BD (1977). Modelling spatial patterns (with discussion).. J R Stat Soc Series B.

[22] Baddeley AJ, Moyeed RA, Howard CV, Boyde A, Down C (1993). Analysis of a three-dimensional point pattern with replication.. Appl Stat.

[23] Rowlingson BS, Diggle PJ (1993). Splancs: Spatial point pattern analysis code in S-plus.. Comput Geosci.

[24] Merton AA, Hoeting JA, Webb CT (2008). Distribution-free comparison of multiple spatial point patterns.. http://www.stat.colostate.edu/research/Technical%20Reports/2008/2008_20.pdf.

[25] Farnsworth ML, Hamilton-West C, Fitchett S, Newman SH, de La Rocque S (2010). Comparing national and global data collection systems for reporting, outbreaks of H5N1 HPAI.. Prev Vet Med.

[26] Rushton J, Viscarra R, Bleich EG, McLeod A (2005). Impact of avian influenza outbreaks in the poultry sectors of five South East Asian countries (Cambodia, Indonesia, Lao PDR, Thailand, Viet Nam) outbreak costs, responses and potential long term control.. Worlds Poult Sci J.

[27] Tiensin T, Ahmed SS, Rojanasthien S, Songserm T, Ratanakorn P (2009). Ecologic risk factor investigation of clusters of avian influenza A (H5N1) virus infection in Thailand.. J Infect Dis.

[28] Gilbert M, Xiao X, Pfeiffer DU, Epprecht M, Boles S (2008). Mapping H5N1 highly pathogenic avian influenza risk in Southeast Asia.. Proc Natl Acad Sci U S A.

[29] Stohr K (2005). Avian influenza and pandemics–research needs and opportunities.. N Engl J Med.

[30] Zepeda C, Salman M, Thiermann A, Kellar J, Rojas H (2005). The role of veterinary epidemiology and veterinary services in complying with the World Trade Organization SPS agreement.. Prev Vet Med.

[31] Vallat B, Pinto J, Schudel A (2006). International organisations and their role in helping to protect the worldwide community against natural and intentional biological disasters.. Rev Sci Tech.

[32] Hadorn DC, Haracic SS, Stärk KD (2008). Comparative assessment of passive surveillance in disease-free and endemic situation: Example of Brucella melitensis surveillance in Switzerland and in Bosnia and Herzegovina.. BMC Vet Res.

